# Using Electronic Health Records for Personalized Dosing of Intravenous Vancomycin in Critically Ill Neonates: Model and Web-Based Interface Development Study

**DOI:** 10.2196/29458

**Published:** 2022-01-31

**Authors:** Ka Ho Matthew Hui, Hugh Simon Lam, Cheuk Hin Twinny Chow, Yuen Shun Janice Li, Pok Him Tom Leung, Long Yin Brian Chan, Chui Ping Lee, Celeste Lom Ying Ewig, Yin Ting Cheung, Tai Ning Teddy Lam

**Affiliations:** 1 School of Pharmacy Faculty of Medicine The Chinese University of Hong Kong Hong Kong Hong Kong; 2 Department of Paediatrics Faculty of Medicine The Chinese University of Hong Kong Hong Kong Hong Kong; 3 Department of Pharmacy Prince of Wales Hospital Hospital Authority Hong Kong Hong Kong

**Keywords:** digital health, web-based user interface, personalized medicine, dose individualization, therapeutic drug monitoring, Bayesian estimation, antibiotics, vancomycin, infectious disease, neonate

## Abstract

**Background:**

Intravenous (IV) vancomycin is used in the treatment of severe infection in neonates. However, its efficacy is compromised by elevated risks of acute kidney injury. The risk is even higher among neonates admitted to the neonatal intensive care unit (NICU), in whom the pharmacokinetics of vancomycin vary widely. Therapeutic drug monitoring is an integral part of vancomycin treatment to balance efficacy against toxicity. It involves individual dose adjustments based on the observed serum vancomycin concentration (VC_s_). However, the existing trough-based approach shows poor evidence for clinical benefits. The updated clinical practice guideline recommends population pharmacokinetic (popPK) model–based approaches, targeting area under curve, preferably through the Bayesian approach. Since Bayesian methods cannot be performed manually and require specialized computer programs, there is a need to provide clinicians with a user-friendly interface to facilitate accurate personalized dosing recommendations for vancomycin in critically ill neonates.

**Objective:**

We used medical data from electronic health records (EHRs) to develop a popPK model and subsequently build a web-based interface to perform model-based individual dose optimization of IV vancomycin for NICU patients in local medical institutions.

**Methods:**

Medical data of subjects prescribed IV vancomycin in the NICUs of Prince of Wales Hospital and Queen Elizabeth Hospital in Hong Kong were extracted from EHRs, namely the Clinical Information System, In-Patient Medication Order Entry, and electronic Patient Record. Patient demographics, such as body weight and postmenstrual age (PMA), serum creatinine (SCr), vancomycin administration records, and VC_s_ were collected. The popPK model employed a 2-compartment infusion model. Various covariate models were tested against body weight, PMA, and SCr, and were evaluated for the best goodness of fit. A previously published web-based dosing interface was adapted to develop the interface in this study.

**Results:**

The final data set included EHR data extracted from 207 subjects, with a total of 689 VC_s_ measurements. The final model chosen explained 82% of the variability in vancomycin clearance. All parameter estimates were within the bootstrapping CIs. Predictive plots, residual plots, and visual predictive checks demonstrated good model predictability. Model approximations showed that the model-based Bayesian approach consistently promoted a probability of target attainment (PTA) above 75% for all subjects, while only half of the subjects could achieve a PTA over 50% with the trough-based approach. The dosing interface was developed with the capability to optimize individual doses with the model-based empirical or Bayesian approach.

**Conclusions:**

Using EHRs, a satisfactory popPK model was verified and adopted to develop a web-based individual dose optimization interface. The interface is expected to improve treatment outcomes of IV vancomycin for severe infections among critically ill neonates. This study provides the foundation for a cohort study to demonstrate the utility of the new approach compared with previous dosing methods.

## Introduction

### Intravenous Vancomycin

Intravenous (IV) vancomycin has long been the first-line treatment for severe bacterial infections, especially in cases involving *Staphylococci* species [1]. Despite its well-established efficacy, vancomycin has a narrow therapeutic index and is commonly associated with acute kidney injury (AKI), especially at high levels of exposure [2]. It was shown that even small acute increases in serum creatinine (SCr) could be detrimental to long-term survival in critically ill patients [3]. Therapeutic drug monitoring (TDM) for vancomycin is a recommended practice to balance efficacy and the risk of AKI. This involves the monitoring of the systemic serum vancomycin concentration (VC_s_) over time after drug administration and subsequent adjustments of vancomycin dosage as necessary.

### Pharmacokinetics of Vancomycin

Vancomycin is eliminated from the systemic circulation primarily through glomerular filtration in the kidneys. Thus, glomerular filtration rate (GFR) is closely correlated with vancomycin clearance (CL), which is the main factor affecting VC_s_ [4]. Since GFR is clinically estimated by creatinine clearance, the major determinants of creatinine clearance, including body size and SCr, are among the major covariates of CL among patients from all age groups [5,6]. To improve the prediction of VC_s_, the pharmacokinetics of vancomycin has been widely studied to understand the mathematical relationship between CL and these covariates [7-11].

### Vulnerability of Critically Ill Neonates Requiring Vancomycin

In the neonatal intensive care unit (NICU), the pharmacokinetics of vancomycin among neonates is highly variable due to dynamic patient conditions and interventions [11]. Moreover, for neonates, it is necessary to account for the maturation of renal function, a process unique to the neonatal population that occurs over the first weeks to months postpartum and is associated with postmenstrual age (PMA) [12]. These conditions put NICU patients at a higher risk for suboptimal therapeutic effects of vancomycin and AKI, making accurate TDM of vancomycin indispensable in this population.

### TDM of Vancomycin

A steady-state area under the curve of the VC_s_-time profile (AUC) over 24 hours (AUC_24_) to minimum inhibitory concentration (MIC) ratio (AUC_24_/MIC) of ≥400 hours has been advocated as the primary predictor of vancomycin efficacy [13]. Nevertheless, since AUC estimation requires measuring multiple VC_s_ values, which is often impractical in the clinical setting, the American Society of Health-System Pharmacists, the Infectious Diseases Society of America, and the Society of Infectious Diseases Pharmacists published a consensus report in 2009 recommending the steady-state trough VC_s_ (VC_s,ss,trough_) as a surrogate marker for the AUC target (assuming MIC at 1 mg/L) [13]. However, data on the efficacy and safety profile with this trough-based approach are lacking [14]. On the other hand, there is further evidence supporting AUC_24_/MIC as the pharmacokinetic target. The requirement of multiple VC_s_ measurements could also be resolved by employing the Bayesian approach as supported by recent research [15,16].

In response, the guideline was updated in 2020 jointly by the 3 societies publishing the 2009 report, together with the Pediatric Infectious Diseases Society, giving new recommendations. First, VC_s,ss,trough_ is no longer recommended as a pharmacokinetic target; dose optimization should instead target an AUC_24_/MIC of 400 to 600 hours. Second, the preferred method to estimate individual AUC is to apply Bayesian estimation using 1 trough VC_s_ (VC_s,trough_, presteady-state or steady-state trough) and preferably 1 peak VC_s_ (VC_s,peak_, presteady-state or steady-state peak), based on a population pharmacokinetic (popPK) model for vancomycin. Third, a less preferred method to calculate individual AUC is to use the first-order equations on a set of measured VC_s,ss,trough_ and steady-state peak VC_s_ (VC_s,ss,peak_) values [14]. Recommendations for initial dosing were also revised. A popPK model–based estimation of individual AUC is preferred over using a universal weight-based dosing scheme [17]. As AUC becomes the basis of dose optimization, ensuring a reliable approach for AUC estimation is a prerequisite of dose optimality. Table 1 summarizes the approaches to AUC estimation and hence dose optimization used in this text.

**Table 1:** Summary of approaches to vancomycin dosing.

Dosing approach	Weight-based	Empirical dosing with popPK^a^ parameter estimates	Steady-state trough target	Estimation of AUC^b^ by steady-state peak and trough	Model-based Bayesian optimization
Which dose to guide?	Initial dose	Initial dose	Maintenance dose	Maintenance dose	Maintenance dose
When to use?	Before the first dose	Before the first dose	When VC_s_^c^ measurement is available	When VC_s_ measurement is available	When VC_s_ measurement is available
Required VC_s_ measurements	N/A^d^	N/A	VC_s,ss,trough_^e^	VC_s,ss,trough_ + VC_s,ss,peak_^f^	VC_s,trough_^g^ (+VC_s,peak_^h^)^i^
PK^j^ target	N/A	AUC	VC_s,ss,trough_	AUC	AUC
popPK model-based?	N/A	Yes^k^	N/A	N/A	Yes^k^
Bayesian estimation required?	N/A	N/A	N/A	N/A	Yes
Recommended?	Yes	Yes^l^	No longer	Yes, less preferred	Yes, preferred

^a^popPK: population pharmacokinetic.

^b^AUC: steady-state area under the curve of the serum vancomycin concentration-time profile.

^c^VC_s_: serum vancomycin concentration.

^d^N/A: not applicable.

^e^VC_s,ss,trough_: steady-state trough serum vancomycin concentration.

^f^VC_s,ss,peak_: steady-state peak serum vancomycin concentration.

^g^VC_s,trough_: trough serum vancomycin concentration (presteady-state or steady-state trough).

^h^VC_s,peak_: peak serum vancomycin concentration (presteady-state or steady-state peak).

^i^Preferrably with VC_s,peak_.

^j^PK: pharmacokinetic.

^k^The 2 approaches are collectively called the model-based approaches.

^l^Potentially better compared with the weight-based approach.

### Multifaceted Roles of Digital Health in the TDM of Vancomycin in the NICU Population

The rapid development in digital health has made this study and the proposed clinical improvements possible in multiple ways. They are elaborated in the following paragraphs.

To keep up with the current standard of treatment and given the large variability in NICU patients, separate popPK analyses for vancomycin are required for the local NICU population [18]. However, prospective data collection is often costly and burdensome in the clinical environment, while the unstructured collection of retrospective data is prone to errors. Fortunately, as digital records are becoming vital on the clinical frontline, electronic health records (EHRs) now present extractable information for data analyses [19]. It is now feasible to consolidate data retrieved from multiple EHR sources to reconcile a data set suitable for popPK analyses [20].

Establishing a popPK model is the first step to upgrade the TDM practice for IV vancomycin in the local NICU population. To maximize the utility of the popPK model, it is necessary to enable Bayesian estimation for accurate estimations of individual AUC [14]. Unlike conventional strategies to individual dose optimization by equations and nomograms, which can be carried out manually, Bayesian estimation requires numerical approximation processes that can only be performed digitally using computers.

Putting popPK model–based Bayesian estimation into clinical practice is difficult because most clinicians are not experts in this area. To tackle this, a fully automated web-based interface incorporating a popPK model, a numerical approximation solution to Bayesian estimation, and algorithms for dose optimization would be an ideal tool for clinical use. In contrast with a client-based interface, a web-based interface (1) allows remote access with various browser-enabled devices, including clinical computer workstations, tablets, and smartphones; (2) saves installation issues; and (3) is easier to maintain. Such an interface is designed to guide and validate necessary inputs from clinicians, followed by suggestions of dosing regimens, which are expected to help clinicians decide the optimal treatment plan that can enhance clinical outcomes.

### Summary and Study Objectives

The use of a model-based dosing interface is in its pilot stage in Hong Kong. Neonatal vancomycin is among the first drugs being investigated. Experiences gained in this study are expected to improve IV vancomycin treatment significantly and, perhaps more importantly, lay the foundation for the extraction of popPK data from EHRs and web-based dose optimization interfaces for other drugs with narrow therapeutic indices. In support of its implementation, this study was conducted in local medical institutions to develop the popPK model of vancomycin for NICU patients using real-world data from EHR resources. Besides, a previously reported framework of a web-based interface performing Bayesian estimation and individual dose optimization for the use of high-dose methotrexate in local institutions will be adopted to create the dosing interface for neonatal IV vancomycin.

## Methods

### Study Population, EHR Use, and Data Preprocessing

The study data set consists of all Chinese patients within 1 year of postnatal age (PNA) admitted to the NICUs of Prince of Wales Hospital and Queen Elizabeth Hospital in Hong Kong between January 1, 2016, and December 31, 2017. Each potential subject had to be prescribed IV vancomycin, and have at least one VC_s_ measurement and one SCr measurement in order to be eligible. Subjects with major congenital heart diseases were excluded. Subjects with vancomycin initiated within 7 days of birth were also excluded due to the variable effects of maternal creatinine on the estimation of neonatal renal function. Eligible subjects were identified through the Clinical Data Analysis and Reporting System (CDARS), a database developed and maintained by the Hong Kong Hospital Authority for audit and research purposes. Data of selected subjects were then collected from several in-house EHR platforms, namely the Clinical Information System (CIS), In-Patient Medication Order Entry (IPMOE), electronic Patient Record (ePR), and, whenever necessary, original copies of medical charts. Ethical approval was obtained from the Joint Chinese University of Hong Kong-New Territories East Cluster Clinical Research Ethics Committee (reference number: 2018.094) and Kowloon Central Cluster/Kowloon East Cluster Research Ethics Committee (reference number: KC/KE-18-0096/ER-1) for data collection. Parental consent was not required due to the anonymized and retrospective nature of data collection.

Constant data items collected were sex, birth weight, gestation age, and date of birth. Time-dependent measurements included body weight, VC_s_, and SCr. Dosing records were collected in terms of the dose administered and the infusion rate (assuming constant rate). The date and time tags of all time-dependent measurements and dosing records were also collected. Dosing records were available from IPMOE, while other data items were collected from CIS and ePR.

All VC_s_ records collected over 7 days after the start of the last infusion of vancomycin were removed. All subjects started on vancomycin within the first 7 days of birth were also removed. At each unique time tag of each subject, PMA was calculated as the time difference between the tag and the estimated first day of the last menstrual period of the subject’s mother. SCr was imputed as the previous or next available value, whichever was closer in time. Body weight was imputed by linearly interpolating and extrapolating available values. All VC_s_ values measured during infusion were removed. SCr values below the lower limit of quantification (LLOQ) (ie, 15 μmol/L) were replaced by 7.5 μmol/L. VC_s_ values measured had an LLOQ of 1 mg/L, and records below the LLOQ (below the limit of quantification [BLQ]) were flagged.

### Model Structure and Parameterization

A popPK model adopts the structure of a nonlinear mixed-effect model [21,22]. A 2-compartment infusion model with first-order elimination was applied, for which the pharmacokinetic parameters CL, central volume (V_c_), intercompartmental clearance (Q), and peripheral volume (V_p_) of vancomycin were defined [9]. The between-subject variability (BSV) in CL and the between-occasion variability (BOV, variability in CL in the same subject between episodes) were expressed in terms of the coefficient of variance (CV) (ie, CVCL and CVCL_BOV_, respectively). Both CVCL and CVCL_BOV_ were assumed to follow the log-normal distribution [23]. Residual unexplained variability was described by a combined proportional-additive error model [24].

In building the pharmacokinetic parameter model, allometric scaling was applied to describe the association of CL, V_c_, Q, and V_p_ against body weight using the power function, with fixed exponents of 0.75 and 1, respectively [25]. This was tested against freely estimated exponents (one for CL and Q, and another for V_c_ and V_p_) using the likelihood ratio test. The maturation of renal function was described as a function of PMA and tested against the linear, exponential, first-order, and Hill functions [26-28]. The function that returned the best goodness of fit was chosen. The renal function with respect to SCr was described using the power model [29].

### Parameter Estimation and Model Evaluation

Parameter estimation was executed with NONMEM version 7.4 (Icon plc) using first-order conditional estimation with interaction [30]. BLQ data were handled using the M3 method [31]. Perl-speaks-NONMEM was used to coordinate NONMEM execution [32]. Residual plots, predictive plots, and a prediction-corrected visual predictive check (pcVPC) were generated [33]. Bootstrapping using 1000 resampled data sets was performed to assess the stability of parameter estimates [34]. R and the R package ggplot2 were used for graphics generation [35,36].

### Dose Individualization

Model-based approaches to dose optimization rely on the estimation of pharmacokinetic parameters for a subject based on the verified popPK model as described above. The set of pharmacokinetic parameter estimates are then used to approximate the AUC distributions at different doses, such that the dose at which the probability of attaining an AUC_24_/MIC of 400 to 600 hours (probability of target attainment [PTA]) is maximized (ie, the optimal dose) can be identified by numerical approximation. Practically, the empirical approach helps decide the initial dose, while the Bayesian approach informs dose adjustments afterwards (see Table 1).

### Web-Based Dosing Interface

The web-based dosing interface in this study is designed to perform the model-based approach to dose optimization. The framework of the interface was replicated from that reported in a previous study for the dose adjustments of single-dose high-dose methotrexate in the pediatric population [37]. On performing the Bayesian approach, the interface demonstrated the ability to generate individual estimates of AUC identical to and more efficiently than NONMEM. The interface was modified to adapt to the popPK model for IV vancomycin estimated and verified in this study, enable empirical dose (the first dose) suggestion, and allow dose optimization at various dosing intervals.

## Results

### Data Set Management

**Figure 1:**
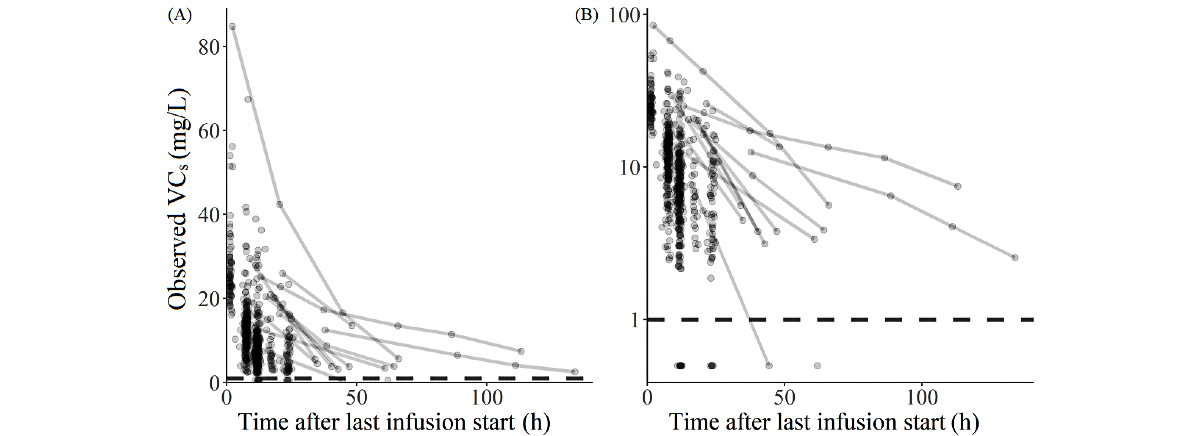
Profile of the observed VC_s_ in the final data set. The graphs show the profile of the observed VC_s_ in linear (A) and logarithmic (B) scales. Observed VC_s_ values after the same last dose in the same subject are joined with a solid line. The dashed horizontal line denotes the lower limit of quantification, below which all measured VC_s_ values are displayed at 0.5 mg/L. VC_s_: serum vancomycin concentration.

One VC_s_ value was measured 7 days after the start of the last infusion and thus removed. Forty-five subjects with PNA <7 days when vancomycin was first started were also removed. Data extraction from the EHRs and data exclusion resulted in a final data set consisting of 207 patients and a total of 689 VC_s_ measurements. The demographics are detailed in Table 2. The time profile of observed VC_s_ values is shown in Figure 1.

**Table 2:** Demographic and data characteristics of the final data set (N=207).

Characteristic		Value
**Site, n (%)**		
	Prince of Wales Hospital	156 (75.4)
	Queen Elizabeth Hospital	51 (24.6)
**Sex, n (%)**		
	Male	112 (54.1)
	Female	95 (45.9)
Gestation age (weeks), median±IQR (min-max)		30.1±6.9 (24.1-41.3)
Postnatal age at first dose (days), median±IQR (min-max)		17±14 (7-114)
Postmenstrual age at first dose (weeks), median±IQR (min-max)		33.7±7.3 (25.7-53.3)
Birth weight (kg), median±IQR (min-max)		1.32±0.89 (0.44-4.14)
Median body weight (kg), median±IQR (min-max)		1.68±1.13 (0.47-7.36)
Dose infused (mg/kg), median±IQR (min-max)		14±3 (5-31)
SCr^a^ (μmol/L), median±IQR (min-max)		42±34 (15-252) (plus 12 BLQ^b^ measures of SCr)
**Number of VC_s_^c^ measurements by subject, n (%)**		
	1	63 (30.4)
	2	43 (20.8)
	3	26 (12.6)
	4	27 (13.0)
	5	21 (10.1)
	6-8	16 (7.7)
	10-21	11 (5.3)
Measured VC_s_ (mg/L), median±IQR (min-max)		9.9±9.4 (1.9-84.8) (plus 16 BLQ measures of VC_s_)
**Number of episodes (after combining) by subject, n (%)**		
	1	131 (63.3)
	2	48 (23.2)
	3	14 (6.8)
	4	5 (2.4)
	5	5 (2.4)
	6	4 (1.9)

^a^SCr: serum creatinine concentration.

^b^BLQ: below limit of quantification.

^c^VC_s_: serum vancomycin concentration.

### Model Comparison

In the final model, a fixed exponent is used for the power functions of the body weight effect and a Hill function is used to describe the PMA-CL relationship. The final model has a minimum objective function value (OFV) of 2329.272. Allowing freely estimated exponents for body weight functions on pharmacokinetic parameters only led to statistically insignificant improvements in goodness of fit (*χ*^2^ value approximates change in OFV [dOFV]=−5.583, *P*=.06, at a degree of freedom of 2). Replacing the Hill function with a linear, exponential, or first-order function resulted in worsened goodness of fit (dOFV=+58.440, +83.391, and +140.645, respectively). None of these alternative models led to significantly better goodness of fit.

### Final Model Parameter Estimates and Evaluation

**Figure 2:**
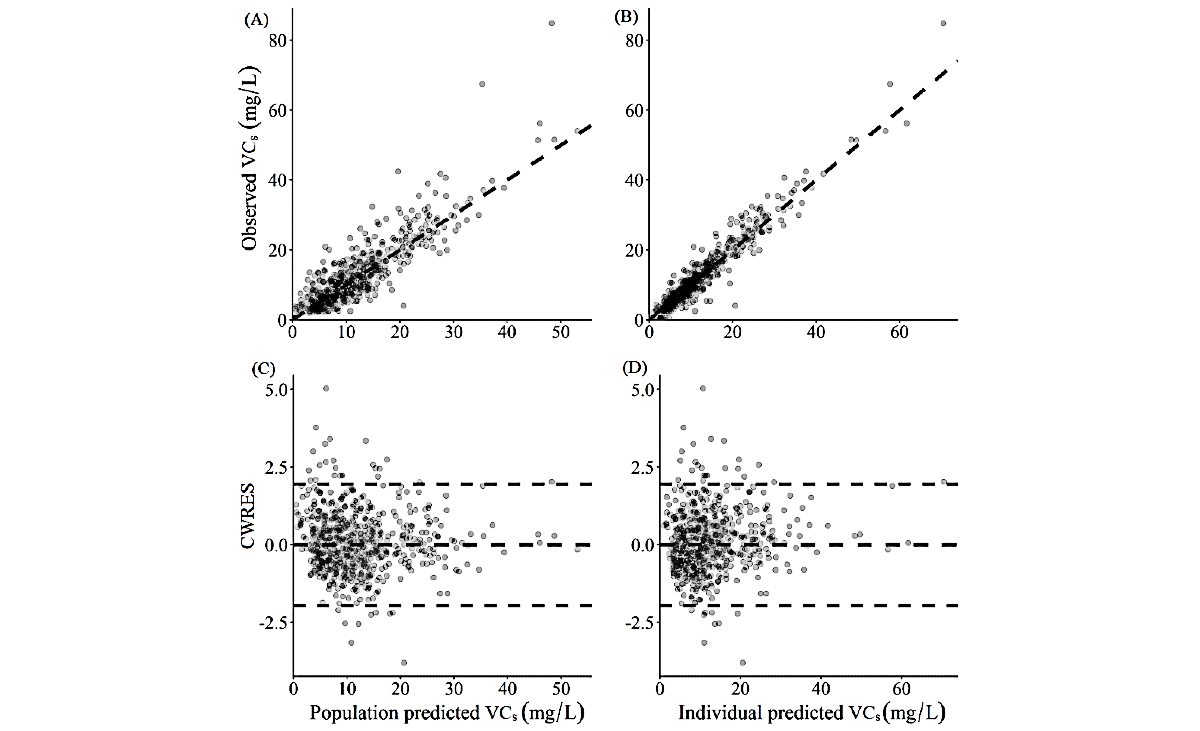
Predictive and residual plots of the final model. The observed VC_s_ and CWRES are plotted against the population and individual predicted VC_s_ of the final model in the graphs, as indicated. The dashed lines in the CWRES plots indicate the range of −1.96 to +1.96, within which 95% of the data points should fall. The observed agreements between observed and predicted VC_s_ and the distributions of CWRES demonstrate the good predictive power of the final model. VC_s_: serum vancomycin concentration; CWRES: conditional weighted residual.

**Figure 3:**
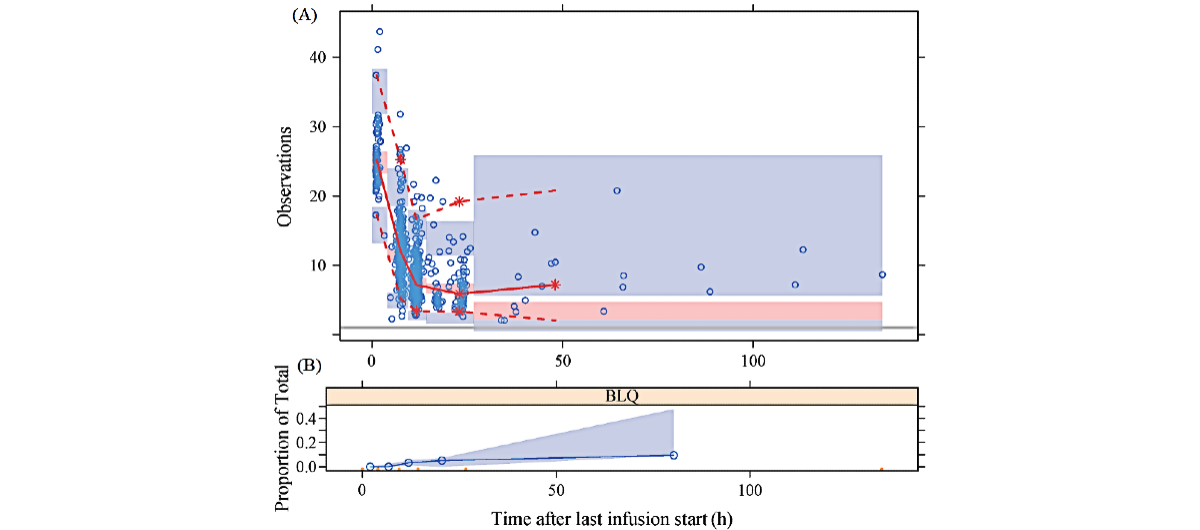
Prediction-corrected visual predictive check of the final model. (A) The 3 shaded areas (from bottom to top) for each time bin represent the 95% CI of the 5th percentiles, medians, and 95th percentiles of the corrected predictions; the dots represent the corrected observed VC_s_; the solid line represents the binned medians of the corrected observed VC_s_; the dashed lines represent the binned 5th and 95th percentiles of the corrected observed VC_s_. Ideally, the percentiles of the observed VC_s_ should fall within the indicated CIs of predicted percentiles. (B) The shaded area and the line represent the 95% CI of predicted proportions and the observed proportions of BLQ concentrations, respectively. Most binned percentiles of the corrected observed VC_s_ fall within or are very close to the 95% CI of corrected predictions, demonstrating the predictive power of the final model. BLQ: below limit of quantification; VC_s_: serum vancomycin concentration.

Parameter estimates for the final model are shown in Table 3. They are very close to the bootstrap means and well within the bootstrap CI and have a condition number of 226 (which is within the usual reference limit of 1000). Accounting for both CVCL and CVCL_BOV_, the effects of body weight, PMA, and SCr alone, and all combined explained 43%, 63%, 54%, and 82% of the variability in CL, respectively. Besides, the overall shrinkage of random effects in CL is estimated to be 16.9%, which is within the acceptable range. Predictive and residual plots of the final model are available in Figure 2. The prediction-corrected visual predictive check is shown in Figure 3.

**Table 3:** Parameter estimates of the final model.

Parameter^a^	Estimate (90% CI)^b^	Bootstrap mean (90% CI)
TVCL^c^, L/h	0.140 (0.123-0.159)	0.142 (0.119-0.163)
*θ*_PMA,CL,Hill_^d^	7.02 (5.05-9.76)	6.76 (5.08-9.70)
*θ*_PMA,CL,Mat50_^e^, days	197 (188-206)	199 (185-209)
*θ*_Scr,CL_^f^	0.541 (0.455-0.644)	0.530 (0.455-0.643)
TVV_c_^g^, L	0.769 (0.705-0.839)	0.782 (0.681-0.868)
TVQ^h^, L/h	0.147 (0.087-0.249)	0.0887 (0.0267-0.8149)
TVV_p_^i^, L	0.285 (0.211-0.385)	0.287 (0.169-0.482)
*θ*_WT,CL_^j^ and θ_WT,Q_^k^	Fixed at 0.75	Fixed at 0.75
*θ*_WT,Vc_^l^ and *θ*_WT,Vp_^m^	Fixed at 1	Fixed at 1
CVCL^n^, %	12.3 (9.0-14.9)	11.9 (8.8-15.0)
CVCL_BOV_^n^, %	13.3 (9.8-16.1)	13.3 (10.4-15.7)
*σ*_prop_^o^, %	16.8 (12.2-23.2)	16.1 (12.0-23.5)
*σ*_add_^p^, mg/L	1.76 (1.25-2.47)	1.68 (1.29-2.40)

^a^The equations for population values are as follows: 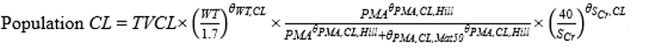; 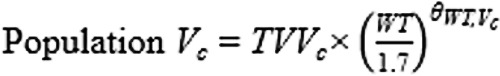; 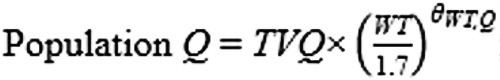; 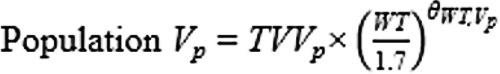, where CL is vancomycin clearance, PMA is postmenstrual age in days, Q is vancomycin intercompartmental clearance, S_Cr_ is serum creatinine level in μmol/L, V_c_ is vancomycin central volume, V_p_ is vancomycin peripheral volume, and WT is body weight in kg.

^b^Parameters were estimated on the logarithmic scale (except for coefficient of variance describing between-subject variability in clearance [CVCL] and coefficient of variance describing between-occasion variability in clearance [CVCL_BOV_]), and the displayed CIs are calculated based on the estimated standard errors on the logarithmic scale assuming normal distribution.

^c^TVCL: typical value of vancomycin clearance.

^d^*θ*_PMA,CL,Hill_: Hill factor describing the association between postmenstrual age in days and vancomycin clearance.

^e^*θ*_PMA,CL,Mat50_: postmenstrual age in days at which maturation in vancomycin clearance is 50%.

^f^*θ*_Scr,CL_: exponent describing serum creatinine effect on vancomycin clearance.

^g^TVV_c_: typical value of vancomycin central volume.

^h^TVQ: typical value of vancomycin intercompartmental clearance.

^i^TVV_p_: typical value of vancomycin peripheral volume.

^j^*θ*_WT,CL_: exponent describing body weight effect on vancomycin clearance.

^k^*θ*_WT,Q_: exponent describing body weight effect on vancomycin intercompartmental clearance.

^l^*θ*_WT,Vc_: exponent describing body weight effect on vancomycin central volume.

^m^*θ*_WT,Vp_: exponent describing body weight effect on vancomycin peripheral volume.

^n^Coefficient of variance describing between-subject variability in vancomycin clearance [CVCL] and coefficient of variance describing between-occasion variability in vancomycin clearance [CVCL_BOV_] are converted from the estimated variance of random effects (*ω*^2^) using the formula 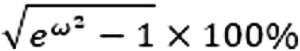.

^o^*σ*_prop_: proportional component of residual unexplained variability.

^p^*σ*_add_: additive component of residual unexplained variability.

### Performance of Dose Individualization

**Figure 4:**
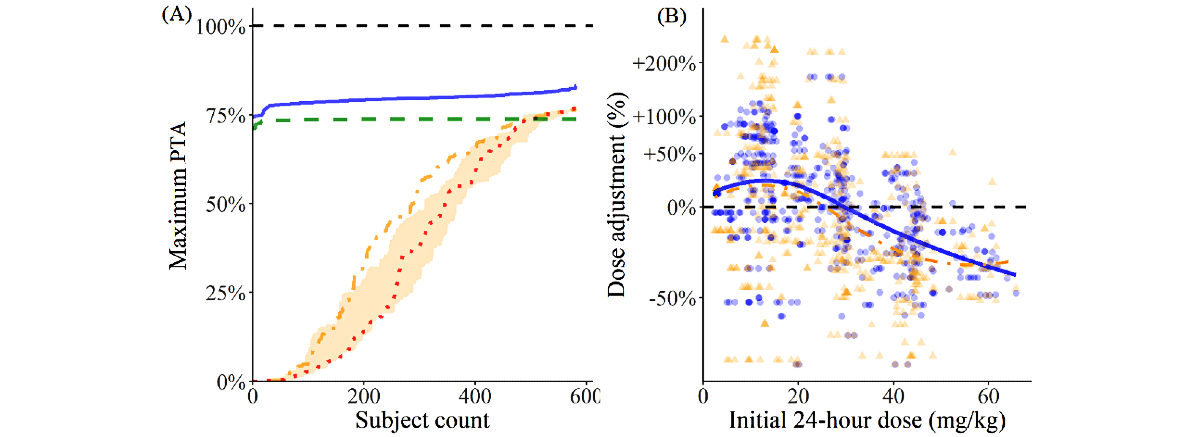
Probability of target attainments with different dose adjustment approaches. (A) The maximum PTA among the indicated count of subjects with the lowest PTAs under different dosing approaches. The lines represent the approximated outcomes of (1) maintaining the initial dose given (red dotted), (2) steady-state trough approach by targeting a VC_s,ss,trough_ of 8.5 mg/L (orange dot-dashed, where the shaded region represents the previously recommended target range of 7-10 mg/L), (3) the model-based empirical approach (green dashed), and (4) the model-based Bayesian approach (blue solid). (B) The percentage changes from the initial 24-hour doses to the optimal doses with the steady-state trough approach (orange triangles with an orange dot-dashed fitting curve) and the model-based Bayesian approach (blue circles with a blue solid fitting curve). The downward sloping fitting curves agree with the general trend that the dose is increased (or decreased) when it is too low (or high). PTA: probability of target attainment. VC_s,ss,trough_: steady-state trough serum vancomycin concentration.

Based on the validated popPK model, the PTAs of different dosing approaches for the subjects in the data set were approximated. The graph on the left in Figure 4 shows that dose adjustments by the steady-state trough approach result in only half of the subjects achieving a PTA over 50%, which is only slightly improved when compared to maintaining the initial doses given. It is also outperformed by the model-based approaches, namely, the empirical approach and the Bayesian approach, which reliably raise the PTA to above 75% for most subjects. Meanwhile, the graph on the right in Figure 4 shows some extreme dose adjustments with the steady-state trough approach when compared to the Bayesian approach, indicating overcorrection of doses with the trough approach without achieving a better PTA profile.

### Web-Based Dosing Interface

**Figure 5:**
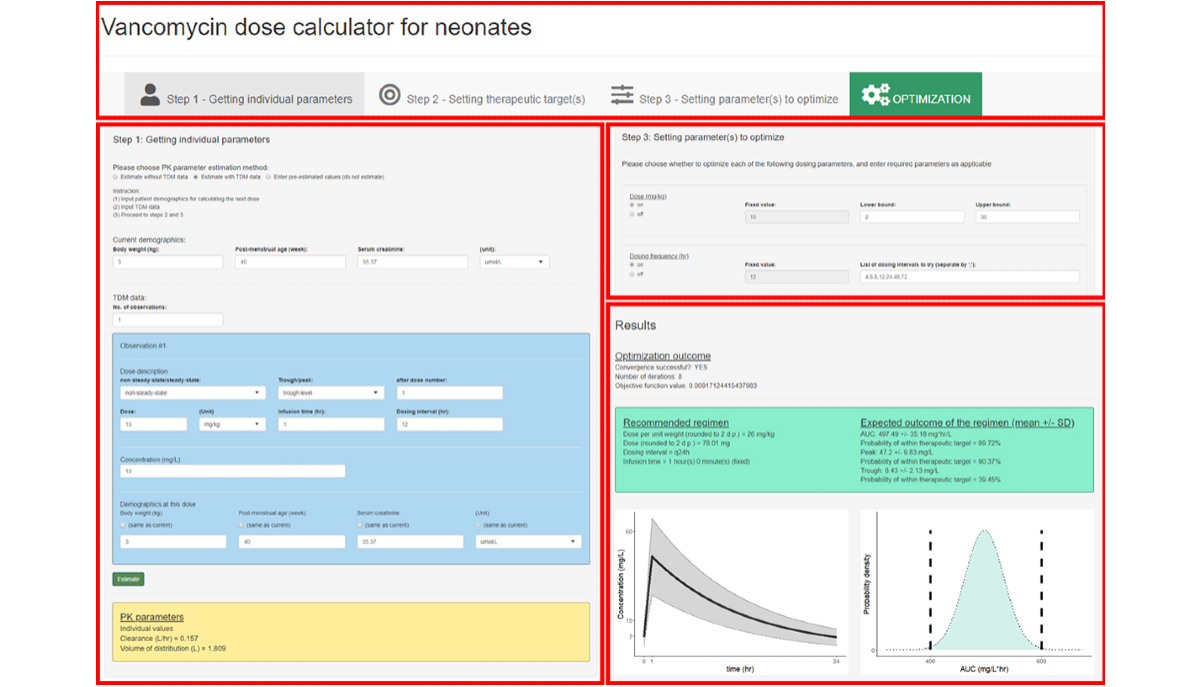
Example screenshot of the individual dose optimization interface. The top part shows the set of ordered tab buttons that are always displayed at the top of the window to guide the users through the steps of using the interface. The left part shows the panel for step 1 to get individual parameter estimates. The upper-right part shows the panel for step 3 to set the ranges of dosing parameters (range of doses and dosing intervals) to optimize. The lower-right part is the panel showing the results of individual dose optimization.

A composite screenshot of the developed interface is available in Figure 5. Detailed screenshots of the developed interface are available in Multimedia Appendix 1. The top panel is always displayed and allows the user to navigate different steps using the interface. By clicking a tab, the corresponding panel will be displayed below the top panel. Step 1 requires user inputs to estimate individual parameters. The user may choose between the model-based approaches (the empirical or Bayesian approach), depending on whether VC_s_ data are available. If the latter is chosen, then apart from the current body weight, PMA, and SCr, the user also needs to input previous doses administered, measured VC_s_, and previous body weight, PMA, and SCr. In step 2, the user may specify the desired therapeutic targets, which defaults to an AUC_24_/MIC of 400 to 600 hours without constraints by VC_s,ss,trough_ and VC_s,ss,peak_. Step 3 allows the user to specify the range of doses and dosing intervals allowed during optimization, which are by default set according to the usual practices of the hospitals using the interface. In most cases, accepting the defaults for steps 2 and 3 suffices. After inputting the required data, the results of individual dose optimization will be generated in the “optimization” tab, which suggests the dose (and dosing intervals) required to maximize PTA and the graphical illustrations of the steady-state VC_s_ profile and the expected probability distribution of AUC_24_.

## Discussion

### Fulfillment of Study Objectives

Real-world data from EHRs were successfully used to develop a popPK model of IV vancomycin for the local NICU population. Based on a previously published dosing interface for high-dose methotrexate, a dosing interface for individual dose optimization of IV vancomycin for the local NICU population was created.

### Reconciliation of popPK Data From EHRs

Despite the promising aspects of using EHRs, challenges were present when attempting to reconcile a popPK data set with EHR resources. First, especially when encountering a new EHR source, efforts were required to understand and validate the data structure of the source to ensure the likeliness of generating necessary tables for statistical analyses. To enable popPK analyses, it is essential to ensure that the target information can be reformatted into tables with different row representations (ie, 1 row per, for example, subject, dose, and observed VC_s_). Then, since EHRs are primarily archived automatically during clinical operation, there is the issue of unstandardized or ambiguous inputs, especially for manual fields, because different clinics may have different logging practices. For instance, laboratories may run assays with different LLOQs, which could be logged onto the EHR systems using various syntaxes. Other problems encountered were suspected duplicated or missing records. For example, detectable VC_s_ measured before the first recorded dose or 7 days after the last dose in subjects with normal renal function may indicate missing dosing records.

A major limitation of using EHR data following the above issues is that data errors and ambiguity are often untraceable. To ensure the robustness of the final data set submitted for popPK analyses, it is crucial to remove problematic data that cannot be clarified from the EHR sources while keeping an eye on the possible risk of causing biased estimates (eg, censored data that are missing not at random).

While having to identify unsalvageable data is a downside, using EHRs is a convenient way to obtain a useful volume of data. Under the hectic environment of hospital wards, it is often difficult for clinicians to cater to the collection of study data. Making use of EHRs can ease the data collection process by minimizing the clinical workforce required. Moreover, since most EHR fields are already standardized, organized, and validated to a certain extent, typographical errors are less of a concern when extracting information from EHR sources.

### popPK Model Development

The covariates can explain a significant proportion of BSV as expected. Diagnostic plots and the prediction-corrected visual predictive check show good predictive performance. The agreement between final parameter estimates against bootstrapping results and the relatively small condition number demonstrates the stability of the estimates. The choices of parameter-covariate relationships in the final model structures and the resultant parameter estimates in this study generally agree with previously reported models [26,28,29]. These positive results of evaluations help establish the validity of the model for implementation into the dose individualization interface.

### Advantages of the Web-Based Dosing Interface

It is anticipated that the implementation of the developed interface can bring about several improvements to the current practice of administering IV vancomycin to treat severe infections in critically ill neonates. First, with the support of the popPK model developed, the interface can estimate individual AUC more accurately and enhance the optimality of the recommended initial dose (with the empirical approach) and maintenance dose (with the Bayesian approach). The recommended dose is also adaptive to significant changes in individual vancomycin PK due to variations in body weight, PMA, and renal functions during treatment. Moreover, with the Bayesian approach, presteady-state VC_s_ is also usable for estimation, such that waiting until the release of a steady-state VC_s_ measurement result for dose adjustment is no longer required. Together, these advantages promote the PTA profile and shorten the time to achieve the pharmacokinetic target by reducing the number of dose adjustments required. This is, in turn, expected to improve the treatment outcomes by promoting recovery while mitigating the risk of developing AKI. Apart from that, implementing the interface eliminates the need for manual calculation and thus reduces the risks of arithmetic errors in dose adjustments. The interface is also designed in a user-friendly and foolproof manner to ease its application by clinicians. Furthermore, the interface is developed using open-source software such that accessibility is guaranteed and licensing costs can be saved.

### Conclusions and Future Studies

Based on a data set reconciled from real-world data extracted from multiple EHR sources, a popPK model of IV vancomycin has been developed and verified for the local NICU population. Based on the verified model and adoption of a previously published framework, a web-based dosing interface has been built to apply model-based approaches to individual AUC estimation and dose optimization of IV vancomycin. The developed interface is expected to improve clinical outcomes of the treatment of severe infections compared with previously adopted approaches, namely, the weight-based approach for initial dosing and the trough-based approach for dose adjustments. A cohort study will be performed later to show the superiority of using the interface compared with the previous approaches in terms of clinical outcomes. The experiences gained in this study will be valuable for the future use of the data collected from EHR sources for popPK analyses and the development of similar interfaces for other drug entities with narrow therapeutic indices.

## Appendix

Multimedia Appendix 1 Detailed screenshots of the web-based dosing interface.

## Abbreviations

AKI: acute kidney injury
AUC: steady-state area under the curve of the serum vancomycin concentration-time profile
AUC_24_: steady-state area under the curve of the serum vancomycin concentration-time profile over 24 hours
BLQ: below the limit of quantification
BOV: between-occasion variability
BSV: between-subject variability
CIS: Clinical Information System
CL: vancomycin clearance
CV: coefficient of variance
CVCL: coefficient of variance of between-subject variability in vancomycin clearance
CVCL_BOV_: coefficient of variance of between-occasion variability in vancomycin clearance
dOFV: change in objective function value
EHR: electronic health record
ePR: electronic Patient Record
GFR: glomerular filtration rate
IPMOE: In-Patient Medication Order Entry
IV: intravenous
LLOQ: lower limit of quantification
MIC: minimum inhibitory concentration
NICU: neonatal intensive care unit
OFV: objective function value
pcVPC: prediction-corrected visual predictive check
PMA: postmenstrual age
PNA: postnatal age
popPK: population pharmacokinetic
PTA: probability of target attainment
Q: vancomycin intercompartmental clearance
SCr: serum creatinine
TDM: therapeutic drug monitoring
V_c_: vancomycin central volume
VC_s_: serum vancomycin concentration
VC_s,peak_: peak serum vancomycin concentration
VC_s,ss,peak_: steady-state peak serum vancomycin concentration
VC_s,ss,trough_: steady-state trough serum vancomycin concentration
VC_s,trough_: trough serum vancomycin concentration
V_p_: vancomycin peripheral volume
