# Supplementary material for: Using Electronic Health Records for Personalized Dosing of Intravenous Vancomycin in Critically Ill Neonates: Model and Web-Based Interface Development Study
Source: JMIR Med Inform. 2022 Jan 31;10(1):e29458. doi: 10.2196/29458 (PMC8844994; doi:10.2196/29458)
Supplement: Multimedia Appendix 1 [file medinform_v10i1e29458_app1.docx]

This is a Multimedia Appendix to a full manuscript published in the Journal of Medical Internet Research. For full copyright and citation information see <http://dx.doi.org/10.2196/jmir.29458>.

This appendix consists of *five* screenshots. Each one shows a part of the web-based dosing interface developed for individual dose optimization of intravenous vancomycin for the treatment of methicillin-resistant *Staphylococcus aureus* among critically ill neonates in Hong Kong:

Figure S1: [Step 1 – Getting individual parameters by estimation without therapeutic drug monitoring (TDM) data (equivalent to the model-based empirical approach stated in the full manuscript)](#A1_1)

Figure S2: [Step 1 – Getting individual parameters by estimation with therapeutic drug monitoring (TDM) data (equivalent to the model-based Bayesian approach stated in the full manuscript)](#A1_2)

Figure S3: [Step 2 – Setting therapeutic target(s)](#A1_3)

Figure S4: [Step 3 – Setting dosing parameter(s) to optimize](#A1_4)

Figure S5: [Result page of optimization](#A1_5)


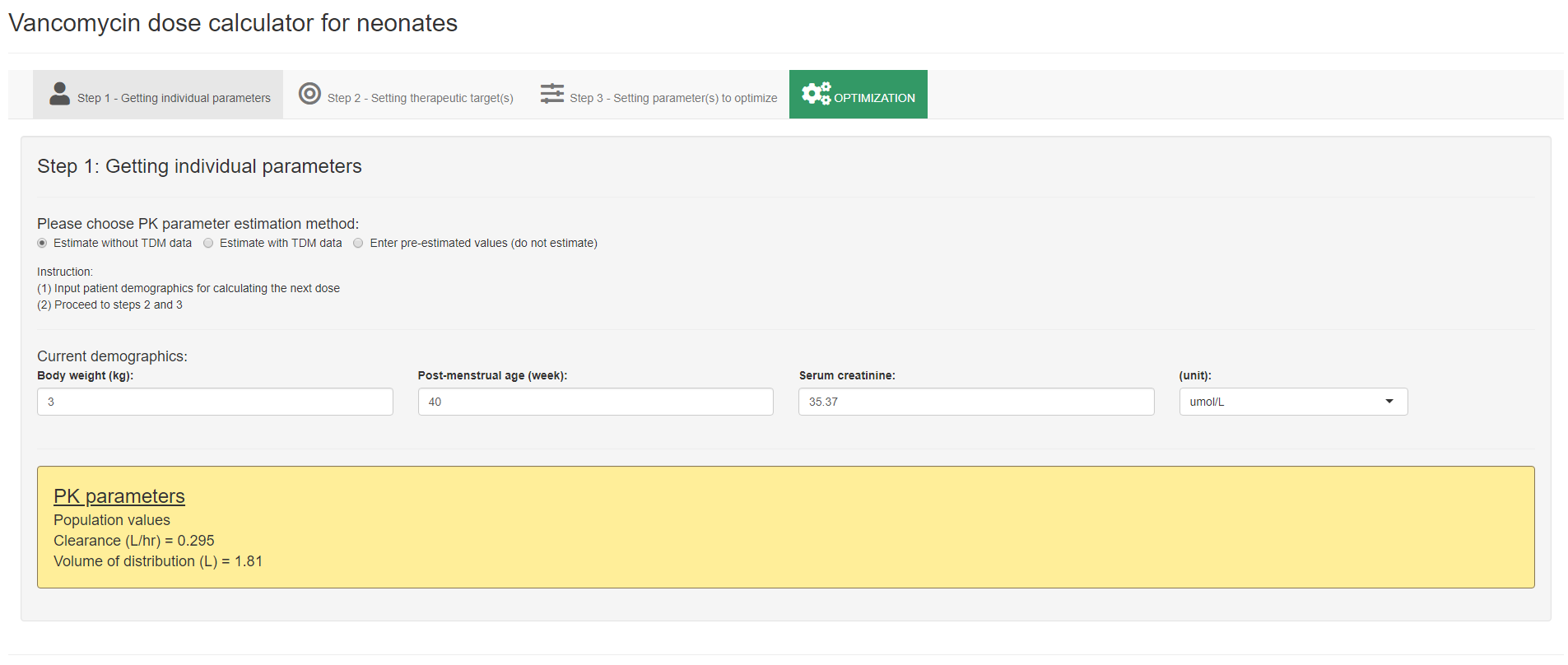


Figure S1: Step 1 – Getting individual parameters by estimation without therapeutic drug monitoring (TDM) data (equivalent to the model-based empirical approach stated in the full manuscript)


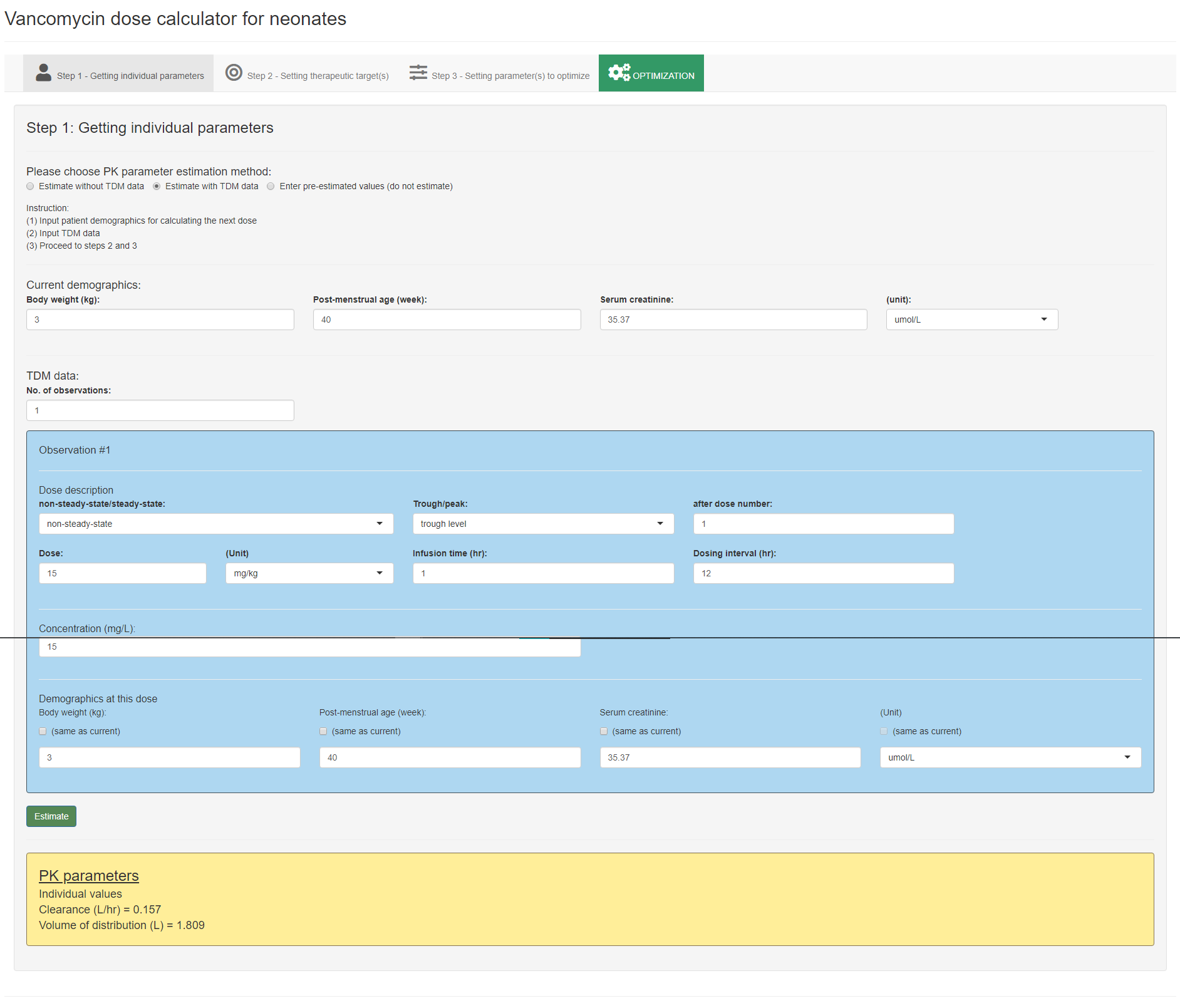


Figure S2: Step 1 – Getting individual parameters by estimation with therapeutic drug monitoring (TDM) data (equivalent to the model-based Bayesian approach stated in the full manuscript)


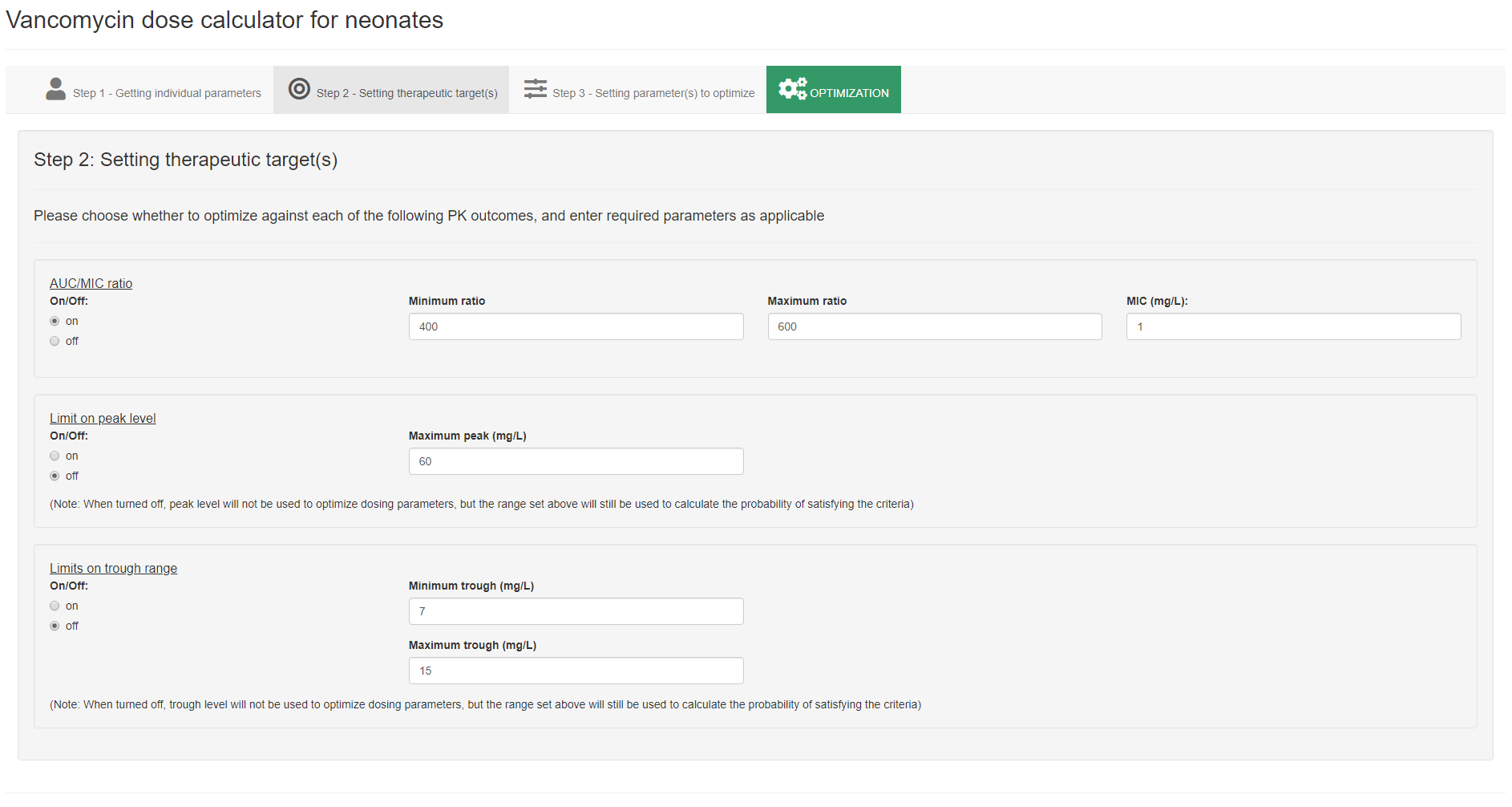


Figure S3: Step 2 – Setting therapeutic target(s)


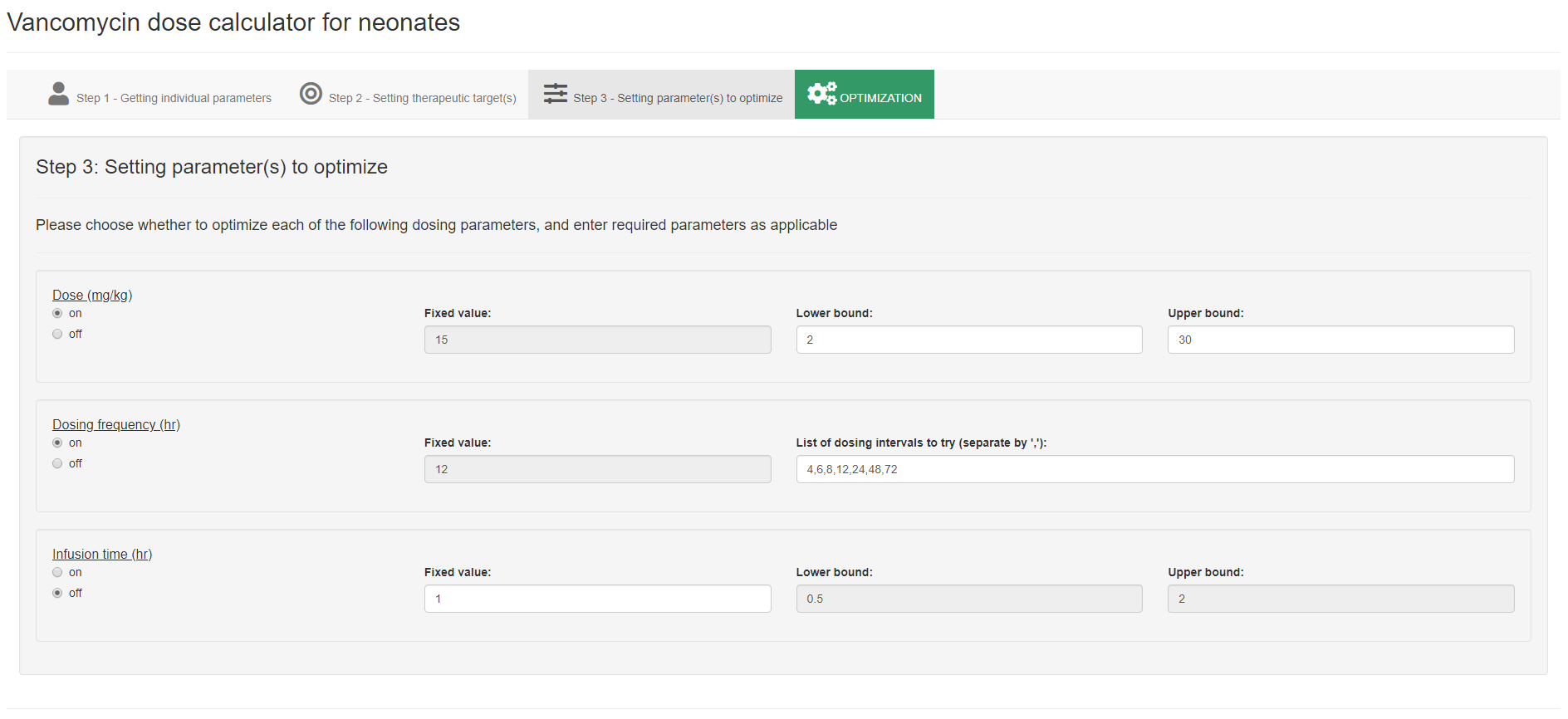


Figure S4: Step 3 – Setting dosing parameter(s) to optimize


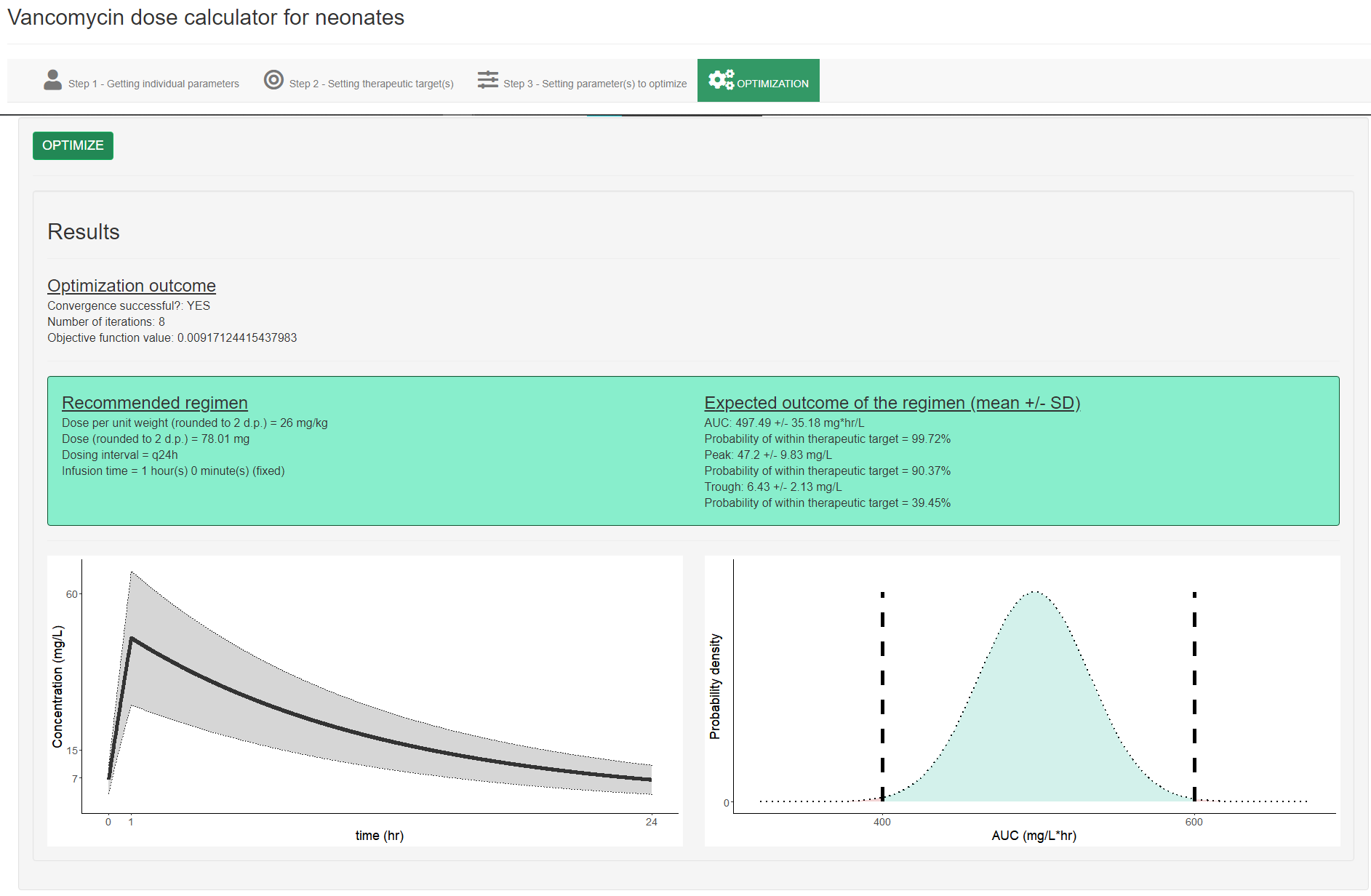


Figure S5: Result page of optimization
